# Outbursts of Passion

**Published:** 1878-02

**Authors:** 


### OUTBURSTS OF PASSION

do certainly tend to shorten life, from the disturbing influence which they have on the circulation, for nature loves regularity and equanimity; this important truth is beautifully demonstrated in the ascertained fact that members of the Society of Friends throughout England, live, on average, twelve years longer than others in the same stations and callings in life, because they are every where a thrifty folk and have a characteristic quietude of deportment and equanimity and serenity of mind, and expression of sentiments which it is beautiful to see, and is really enviable. And so has the immortal Watts sung of those who live under the calming influence of the Christian religion.

Swift as their thoughts, their joys come on,
But fly not half so swift away —
Their souls are ever bright as noon,
And ca.m as summer evenings be
Their days glide sweetly over their heads,
Made up of innocence and love,
And soft and silent as the shades
Their nightly moments gently move.

It is then a legitimate conclusion that the best means of assuring to ourselves a long life of healthfulness and quiet enjoyment is to bring the whole life under the influence of Christian principal, to embark in the making of money in all legitimate honorable ways, since this gives occupation to both mind and body, which might otherwise become depraved through the corroding vice of indolence.

